# Prognostic Factors for Sideroblastic Anemia with B-cell Immunodeficiency, Periodic Fevers, and Developmental Delay Due to *TRNT1* Gene Mutations: A Case Report and Systematic Review

**DOI:** 10.1007/s10875-026-02000-6

**Published:** 2026-03-07

**Authors:** Tzu-Hsuan Su, Ni-Chung Lee, Li-Chieh Wang, Bor-Luen Chiang, Hsin-Hui Yu

**Affiliations:** 1 Department of Pediatrics, National Taiwan University Children’s Hospital, No. 7, Zhongshan S. Road. Zhongzheng District, Taipei City, 100 Taiwan; 2 Department of Medical Genetics, National Taiwan University Hospital, Taipei, Taiwan; 3 Department of Medical Research, National Taiwan University Hospital, Taipei City, 100 Taiwan; 4 Genome & Systems Biology Degree Program, College of Life Science, National Taiwan University, Taipei, Taiwan

**Keywords:** TRNT1, Sideroblastic anemia, B-cell deficiency, Periodic fevers, SIFD

## Abstract

Sideroblastic anemia with B-cell immunodeficiency, periodic fevers, and developmental delay (SIFD), a rare multisystemic syndrome, occurs due to loss-of-function mutations in the tRNA nucleotidyl transferase 1 (*TRNT1*) gene. This study reports the case of a 21-month-old female patient with SIFD and compound heterozygous mutations c.824T > A, p.Leu275X (a novel variant) and c.1246 A > G, p.Lys416Glu in *TRNT1* gene. The patient had presented with recurrent fever since 10 days of age, along with vasculitis, systemic inflammation with elevated proinflammatory cytokines, decreased B-cell count, and failure to thrive. Furthermore, she did not respond to intravenous immunoglobulin (IVIG) treatment, but her condition stabilized with etanercept (a tumor necrosis factor [TNF] inhibitor) and corticosteroids therapy. In addition, this study includes a systematic review of the clinical presentations, genetic mutations, treatments, and outcomes of 75 patients with SIFD. The estimated 2-, 5-, and 10-year Kaplan–Meier survival probabilities for all patients were 88.45%, 76.67%, 68.84% for all patients; 82.40%, 58.86% and 44.85% for patients with onset age of ≤ 3 months; 70%, 40% and 26.68% for patients with seizures; 88.05%, 66.62% and 59.22% for patients with decreased B cell number; 50% and 33.33% for patients who received hematopoietic stem cell transplantation (HSCT), respectively (log-rank *P* < 0.05). We concluded that younger age of onset of ≤ 3 months, seizures, and decreased B-cell count are significant poor prognostic factors for survival. Anti-TNF therapy early in life may stabilize patients with autoinflammatory phenotypes; however, the role of HSCT remains controversial.

**Supplementary Information:**

The online version contains supplementary material available at 10.1007/s10875-026-02000-6.

## Introduction

Sideroblastic anemia with B-cell immunodeficiency, periodic fevers, and developmental delay (SIFD), a rare multisystemic syndrome, occurs due to the loss-of-function mutations in the *TRNT1* gene [1, 2], which encodes CCA tRNA nucleotidyltransferase 1. TRNT1 adds a trinucleotide sequence cytosine–cytosine–adenine (CCA) to the 3ʹ terminus of transfer RNA (tRNA) for correct positioning of tRNA at the ribosome to terminate translation. This post-transcriptional modification is essential for the synthesis of mitochondrial and cytosolic proteins [3].

*TRNT1* mutations negatively affect the expression and function of mature mitochondrial and cytosolic tRNA, leading to the impairment of protein synthesis and clearance via autophagy [4, 5]. Subsequent mitochondrial dysfunction and intracellular or endoplasmic reticulum (ER) stress are associated with autoinflammation [5]. Immunological defects include B cell maturation arrest at the pre-B cell stage, decreased follicular T helper cell count, and impaired antibody production by plasma cells [6, 7].

A wide spectrum of clinical and immunological heterogeneity due to *TRNT1* mutations has been described following the first study on SIFD by Wiseman et al. [8]. Severe impairment of the hematological and immunological systems might result in high morbidity and mortality. In contrast, milder forms may involve moderate anemia, cardiomyopathy, retinitis pigmentosa, and cataracts [7]. This study reports a case of a 21-month-old female patient with SIFD and compound heterozygous *TRNT1* genetic mutations with a novel variant. Notably, fever and systemic inflammation in the patient were well controlled with etanercept, an anti-tumor necrosis factor (anti-TNF) therapy. Additionally, we performed a systematic review of the clinical presentations, genetic mutations, treatments, and outcomes of 75 patients (including the current case) with SIFD to identify significant factors associated with survival and the role of hematopoietic stem cell transplantation (HSCT).

## Methods

### Patient and Healthy Controls

The patient was prospectively enrolled, and healthy controls were recruited for immunological assays. Data regarding clinical manifestations and treatments of the patient, as well as laboratory and immunological data, were collected and analyzed. This study was conducted in accordance with the guidelines of the Declaration of Helsinki, 1964, and was approved by the Institutional Research Ethics Committee of the National Taiwan University Hospital (IRB no. 202112077RIND).

### Genetic Analysis

Genomic DNA was extracted from peripheral blood of the patient and both parents, and whole-exome sequencing (WES) of the patient was performed. Notably, capture-based (Roche KAPA HyperExome) exome sequencing (NovaSeq, Illumina) was performed. Burrows–Wheeler aligner was used for sequence alignment with the human reference genome (Genome Reference Consortium GRCh38). Both mitochondrial and nuclear genomes were analyzed. Genome Analysis Tool Kit (GATK, Broad Institute, US) was employed to perform variant calling. The variants were annotated using Variant Studio and wANNOVAR. Variants with minor allele frequencies of < 0.001 in both the 1000 Genomes and Exome Sequencing Project databases and nonsense, splice, or nonsynonymous mutations were retained for downward analysis. Variant pathogenicity data from the Human Gene Mutation Database, ClinVar, OMIM, and allele frequency data from Taiwan Biobank were added to the annotation file. The pathogenicity of the mutations was assessed according to the American College of Medical Genetics and Genomics guidelines. Genetic variations in *TRNT1* in the patient and their parents were confirmed using Sanger sequencing.

### Literature Review

A literature review of SIFD was performed using the PubMed and EMBASE databases. The search terms were “Sideroblastic anemia with B-cell immunodeficiency, periodic fevers, and developmental delay,” “Sideroblastic anemia AND Immunodeficiency,” “SIFD,” and “TRNT1.” Studies, including articles, reviews, letters, case reports, or meeting abstracts, published online in English before November 30, 2023, with results containing genetic mutations or clinical characteristics and outcomes, were included in the review.

### Statistical Analysis

Descriptive statistics are expressed as medians with interquartile ranges or ranges for continuous variables and as numbers (percentages) for nominal variables. Fisher’s exact test or the chi-square test were used to compare differences between groups for categorical variables, whereas Mann–Whitney U test was used for continuous variables. Kaplan–Meier analysis was used to analyze the survival, and log-rank tests were performed for comparison. All data were analyzed using GraphPad Prism (GraphPad Software, San Diego, CA, USA) and SAS software (version 9.4; SAS Institute, Inc., Cary, NC, USA). Statistical significance was set at two-tailed *P* < 0.05.

## Results

### Case Report

**Fig. 1:**
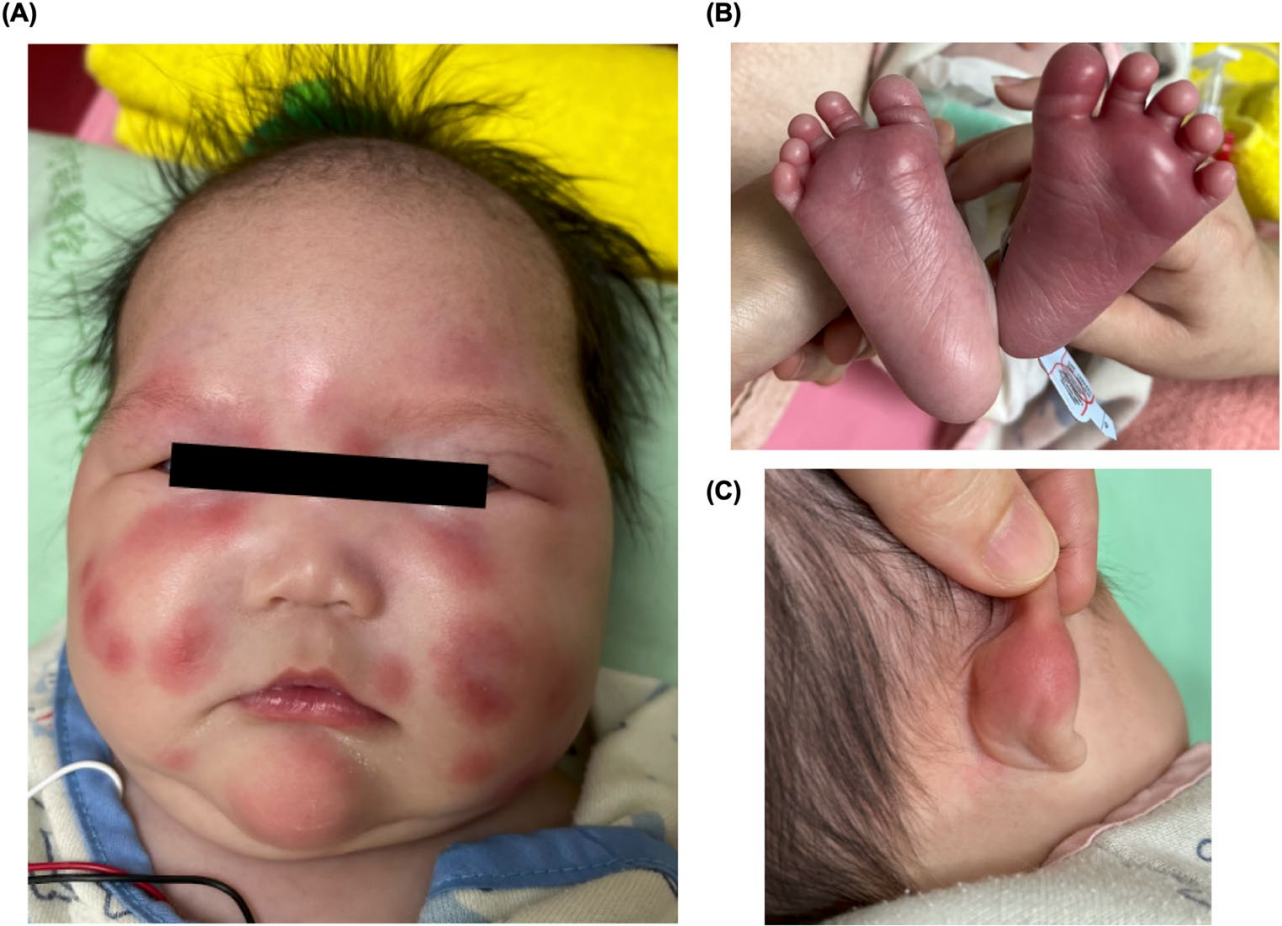
Photography of the patient. Red-to-purple papules with local swelling over the face, ears, and feet of the patient with *TRNT1* mutation at an age of 1 month

A 21-month-old female patient included in this study was born to nonconsanguineous parents. She developed a recurrent fever at 10 days of age, which occurred every 1–2 weeks. Notably, red-to-purple papules with local swelling appeared on her face and limbs during periods of fever (Fig. 1). The patient received high-dose intravenous immunoglobulin (IVIG) therapy and oral corticosteroids because of the suspicion of atypical Kawasaki disease.

The patient was transferred to our hospital at 3 months of age. Cardiac echocardiography showed no dilatation; however, the loss of tapering and perivascular brightness of the coronary arteries indicated atypical Kawasaki disease. The infection analysis did not reveal any evidence of pathogens, including culture or polymerase chain reaction [PCR] detection from blood, sputum, urine, stool, and cerebrospinal fluid for bacterial, fungal, viral, atypical or opportunistic pathogens. There was no definite active inflammation was identified on the 2-deoxy-2[18 F]fluoro-d-glucose (FDG) positron emission tomography/computed tomography.

Immunological profiling revealed decreased B cell count (370.2 cells/µL, reference range 500–1500 cells/µL) and serum IgM level (20.64 mg/dL, reference range 24–89 mg/dL). Laboratory data showed considerably increased serum levels of C-reactive protein (> 40 mg/dL, reference range < 1 mg/dL), erythrocyte sedimentation rate (58 mm/hr, reference range 2–20 mm/hr), procalcitonin (13.21 ng/mL, reference range < 0.5 ng/mL), ferritin (3593.44 mg/dL, reference range 4.63–204 mg/dL), TNF-α (156 pg/mL, reference range < 8.1 pg/mL), and IL-6 (694.3 pg/mL, reference range < 7.0 pg/mL) during febrile episodes (Supplementary Table S1). The serum iron and total iron binding capacity were 61 µg/dL (reference range 51–209 µg/dL) and 282 µg/dL (reference range 268–593 µg/dL), respectively.

**Fig. 2:**
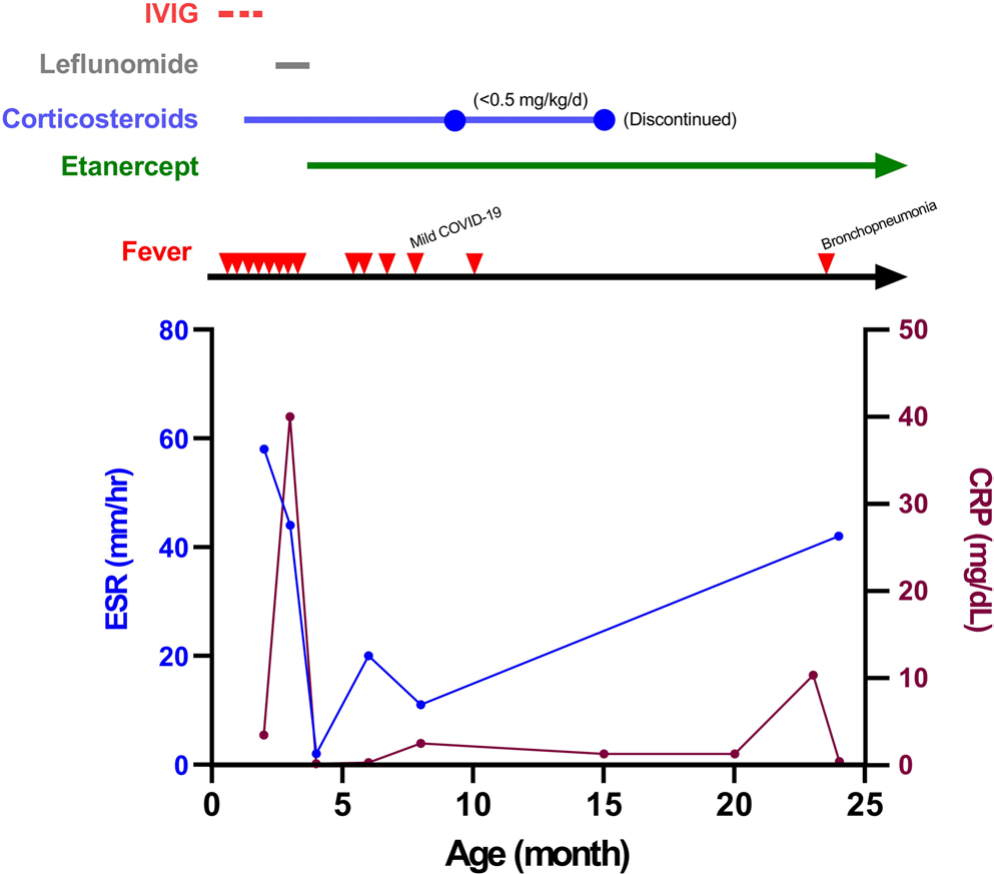
Schematic diagram of febrile episodes, inflammatory markers and treatment. The febrile episodes are denoted as red inverted triangles. CRP, C-reactive protein; ESR, erythrocyte sedimentation rate; IVIG, intravenous immunoglobulin; COVID-19, coronavirus disease 2019

Trio whole-exome sequencing (WES) was performed on the patient and her parents. Compound heterozygous *TRNT1* gene mutations—c.824T > A, p.Leu275X (a novel variant inherited from her father) and c.1246 A > G, p.Lys416Glu (inherited from her mother, pathogenic)—were identified in the patient (Supplementary Table S2). Under the diagnosis of SIFD syndrome, the patient was administered oral prednisolone (2 mg/kg/day), gradually tapering to a low maintenance dose, followed by weekly subcutaneous injections of etanercept (0.8 mg/kg/week)—a soluble TNF receptor fusion protein—at 4 months of age [5, 9]. The fever, vasculitis rash, and swelling of the bilateral knee joints were well controlled. Additionally, bilateral sensorineural hearing impairment was confirmed using an auditory brainstem response test, and failure to thrive and developmental delay were observed during the follow-up and rehabilitation at our hospital. A schematic diagram of febrile episodes, inflammatory markers and treatment for our patient is shown in Fig. 2.

### Systematic Review

**Fig. 3:**
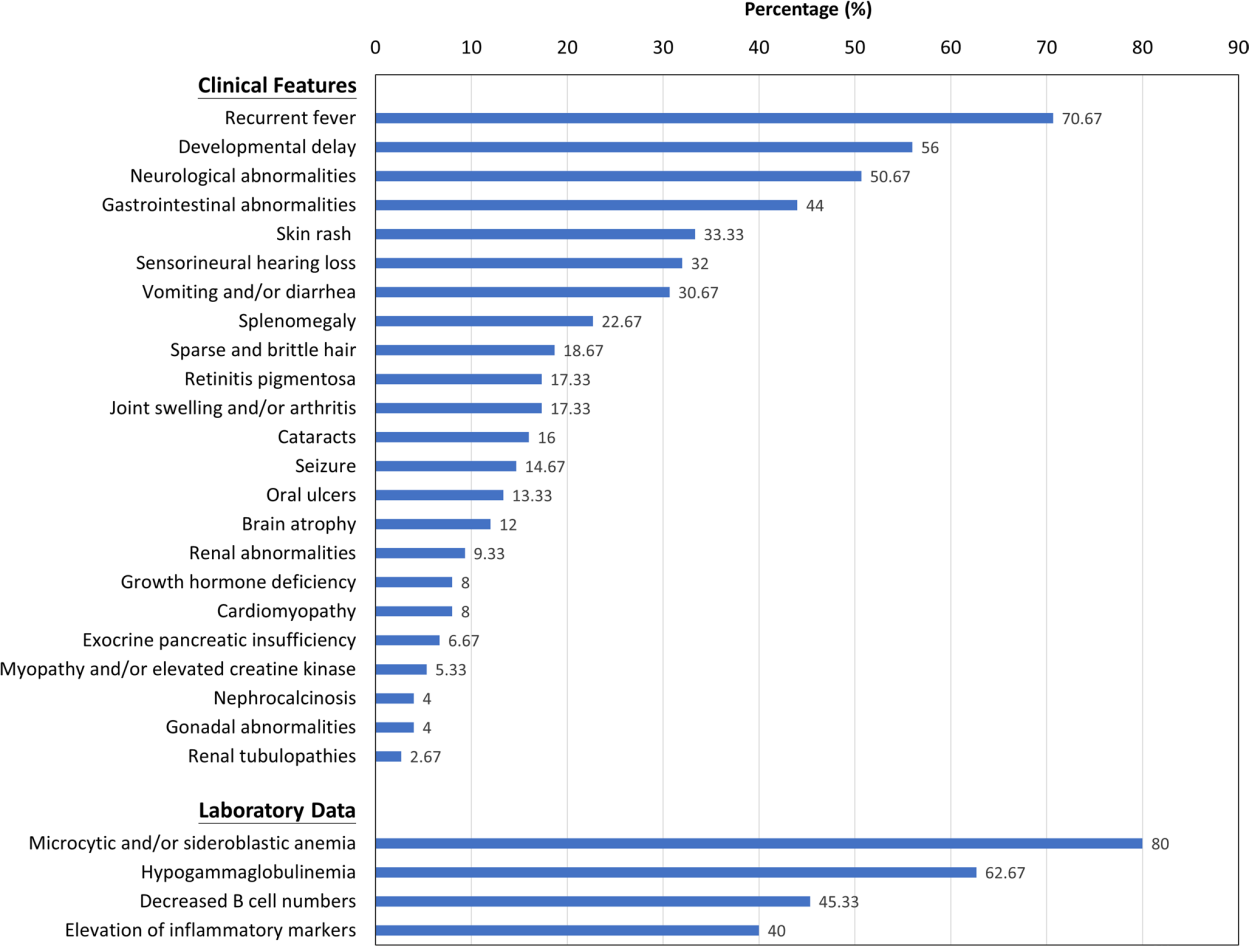
Clinical and laboratory features in 75 patients with *TRNT1* mutations

Seventy-five patients with SIFD, including our patient, have been reported in English literature (Table 1; Fig. 3 and Supplementary Table S3) [1, 2, 4, 5, 7–10, 13–40]. Common clinical presentations included anemia (80%), recurrent fever (70.67%), hypogammaglobulinemia (62.67%), developmental delay (56%), and gastrointestinal abnormalities (44%). B-cell lymphopenia and/or antibody deficiencies may develop later in life [8].

**Table 1:** Demographic data, treatment, and outcomes in 75 patients with *TRNT1* mutations

	Patients (*N* = 75)*N* (%)
Female	36/69 (47.8%)
Age of onset (months) (median, IQR)	4 (1.5-8)
Treatment	
Intravenous and/or subcutaneous immunoglobulins	37 (49.33%)
Systemic corticosteroids	15 (20%)
Anti-TNF therapy	11 (15.67%)
Disease-modifying antirheumatic drugs	7 (9.3%)
Anti-IL-1 therapy (anakinra)	6 (8%)
Hematopoietic stem cell transplantation	6 (8%)
Outcomes	
Death	21/74 (28.38%)
Age of death (years) (median, IQR)	2.17 (1.17-4.83)
Age at the last follow-up (years) (median, IQR)	5.0 (2.67-12.5)

*TNF* tumor necrosis factor, *IL-1* interleukin-1, *IQR* interquartile range

Disease-modifying antirheumatic drugs: 4 colchicine, 1 methotrexate, 1 azathioprine, 1 thalidomide

### Genetic Mutations

**Fig. 4:**
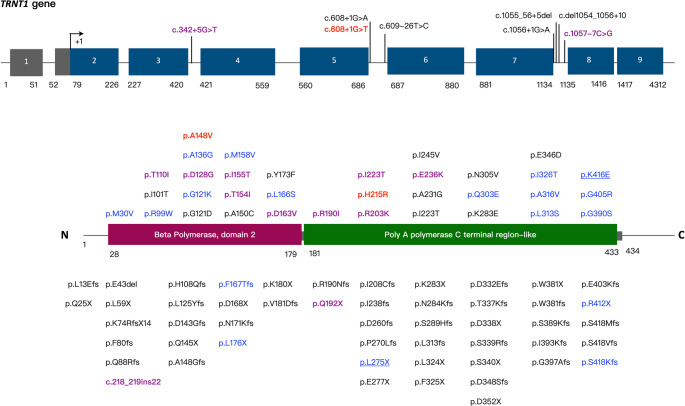
Genetic mutations in *TRNT1*. Genetic variants of *TRNT1* reported in 75 patients and in the Genome Aggregation Database (gnomAD) are shown as splice mutations in the upper part, missense variants in the middle part, nonsense and frameshift mutations in the lower part of the Figure. TRNT1 protein consists of two domains: beta polymerase, domain 2, and poly A polymerase C terminal region-like according to the InterPro database (https://www.ebi.ac.uk/interpro). Genetic variants are shown in red (death without B cell deficiency), purple (death with B cell deficiency), blue (alive with B cell deficiency), or black (alive without B cell deficiency). The genetic variants in our patient are underlined

Figure 4 presents the 92 genetic variants in *TNRT1*. Notably, 19 patients presented with homozygous mutations, whereas 51 exhibited compound heterozygous mutations and 3 were heterozygous with single allelic mutation. The reported *TNRT1* mutations among 138 alleles were missense (98, 71.01%), splicing (19, 13.77%), frameshift (14, 10.14%), nonsense (6, 4.35%), and deletion (1, 0.72%). The most frequent variants were c.668T > C, p.Ile223Thr (missense, 19 alleles, 13.77%), c.295 C > T, p.Arg99Trp (missense, 13 alleles, 9.42%), c.1246 A > G, p.Lys416Glu (missense, 10 alleles, 7.24%), and c.1057–7 C > G (splicing, 8 alleles, 5.8%). The variants associated with death are marked in red (without B cell deficiency) or purple (with B cell deficiency) in Fig. 4, and c.668T > C, p.Ile223Thr (missense, 9 deceased/17 patients with mutated alleles) and c.608 + 1G > T (splicing, 3 deceased/3 patients with mutated alleles) mutations were frequently observed in patients who died. Mutations located in the C-terminal half of the TRNT1 protein were associated with better survival (93.6%, 44 survived/47 mutations) compared with mutations occurring in the N-terminal half (71.1%, 32 survived/45 mutations). Types of mutations (nonsense/missense/frameshift/splice) did not correlate with survival or B cell deficiency (Supplementary Table S4). Notably, the nonsense variant c.824T > A, p.Leu275X in our patient is a novel mutation that has not been reported previously.

### Treatment and Outcomes

The 75 patients with SIFD were treated with IVIG and/or subcutaneous immunoglobulins (SCIG) (49.33%), systemic corticosteroids (20%), anti-TNF therapy (adalimumab, infliximab, and etanercept) (15.67%), disease-modifying antirheumatic drugs (9.3%), and anti-IL-1 therapy (anakinra) (8%) (Table 1). The mortality rate was 21/74 (28.38%), with the median age of death and median age at the last follow-up being 2.17 years (range: 0.01–14 years) and 5.0 years (range: 40 h–49 years) (Table 1), respectively. Of the six patients who received HSCT, four patients (66.67%) died at a median age of 1.8 years (range: 0.5–3 years). Notably, all 11 patients who received anti-TNF therapy survived, with a median age of 4.7 years (range: 1.8–13 years), and demonstrated clinical improvement: 11 with less febrile episodes, 10 with normalization of inflammatory markers, 6 with improvement of anemia, 3 with resolution of skin symptoms, and 1 with reduction of seizures.

**Fig. 5:**
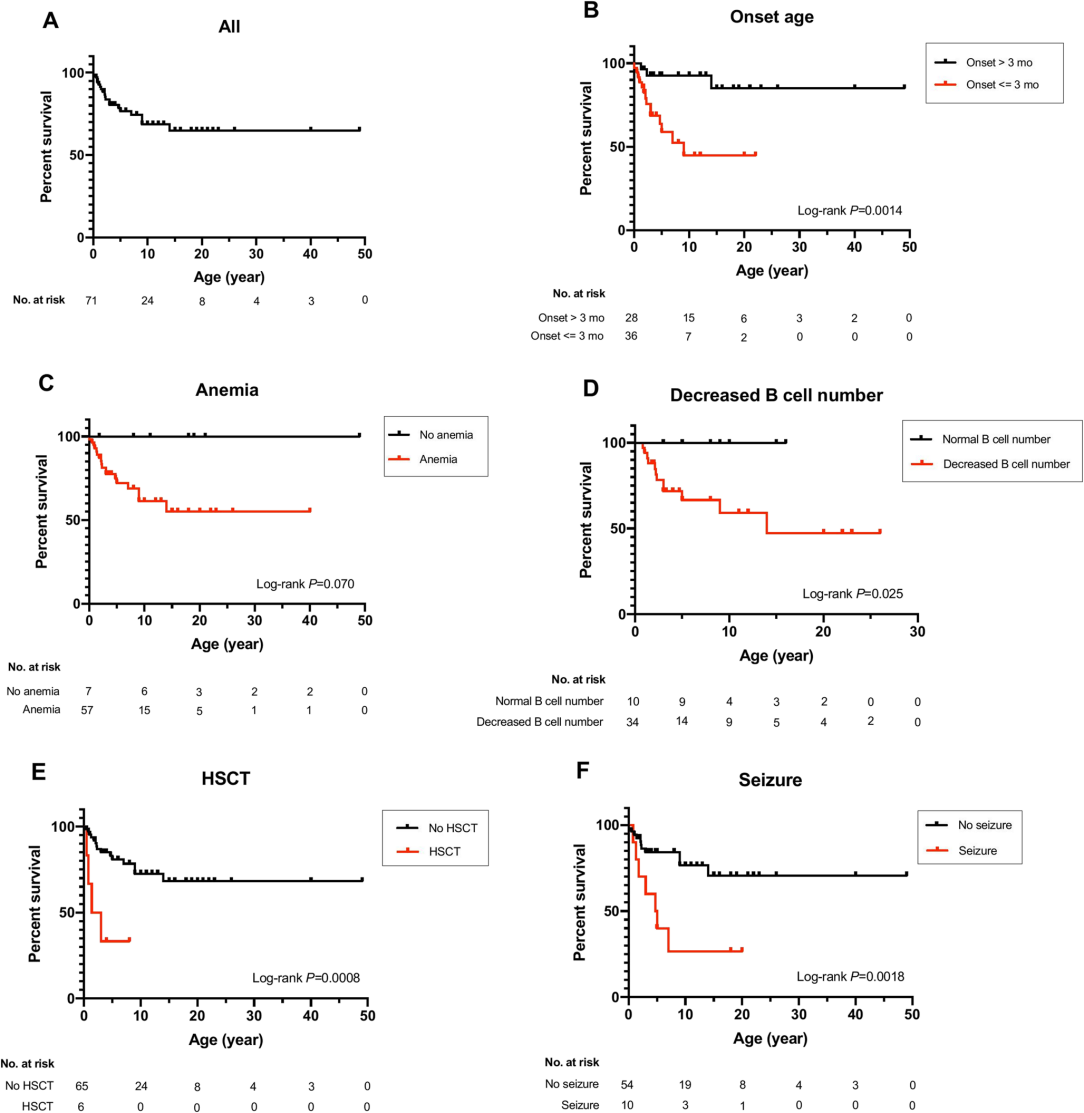
Survival analysis. Kaplan–Meier survival curves for overall survival in all patients with *TRNT1* mutations (**a**), in patients with onset age of ≤ and > 3 months (**b**), in patients with and without anemia (**c**), in patients with and without decreased B cell count (in patients with available data on B cell count) (**d**), in patients with and without hematopoietic stem cell transplantation (HSCT) (**e**), and in patients with and without seizures (**f**). The comparison of survival between two groups with the log-rank *P* value is shown

A higher proportion of patients who died exhibited an onset age of ≤ 3 months (82.35% vs. 46.81%, *P* = 0.021), a decreased B cell count (100% vs. 68.75%, *P* = 0.041), seizures (38.89% vs. 6.52%, *P* = 0.004), and received HSCT (21.05% vs. 3.85%, *P* = 0.04) than the patients who were alive (Supplementary Table S5). The estimated 2-, 5-, and 10-year Kaplan–Meier survival probabilities for all patients were 88.45%, 76.67%, 68.84% for all patients; 82.40%, 58.86% and 44.85% for patients with onset age of ≤ 3 months; 70%, 40% and 26.68% for patients with seizures; 88.05%, 66.62% and 59.22% for patients with decreased B cell number; 50% and 33.33% for patients who received HSCT, respectively. Survival probabilities significantly decreased in patients with onset age ≤ 3 months (log-rank *P* = 0.0014), seizures (log-rank *P* = 0.0018), decreased B cell count (log-rank *P* = 0.025), or those received HSCT (log-rank *P* = 0.0008) (Fig. 5). For the two patients with monoallelic mutation and detailed clinical information, patient 5 and 54 carried two risk parameters: onset age ≤ 3 months and decreased B-cell count for patient 5; and ≤ 3 months and seizures for patient 54 (Supplementary Table S3).

## Discussion

Sideroblastic anemia with B-cell immunodeficiency, periodic fevers, and developmental delay (SIFD) is a multisystemic disease characterized by congenital sideroblastic anemia (pathological iron accumulation in the mitochondria of red blood cell precursors in the bone marrow) and periodic fever. Our findings revealed an association of *TRNT1* mutations located near the C-terminus with survival (Fig. 4). Although mitochondrial dysfunction plays an important role in SIFD pathogenesis, the underlying mechanisms remain largely unknown. Some studies demonstrated impaired respiration and composition of oxidative phosphorylation complexes in patients with SIFD [4, 41]. Fatica et al. showed that *TRNT1* mutations may be associated with increased reactive oxygen species production and sensitivity to oxidative stress, which is due to exacerbated, angiogenin-dependent cleavage of tRNAs [42]. Furthermore, the high clinical variability of SIFD indicates the role for other modifying factors or genes.

Our patient exhibited typical features of systemic autoinflammation, including fever, vasculitis rash, elevated levels of inflammatory mediators, and failure to thrive, but did not present with significant anemia, immunodeficiency, or recurrent infections. The study by Giannelou et al. demonstrated that increased mitochondrial reactive oxygen species, persistent NOD-, LRR-, and pyrin domain-containing protein 3 inflammasome activation, and clinical response to anti-IL-1 therapy (anakinra) were observed in a few patients with SIFD [5, 9, 10].

Intravenous immunoglobulin (IVIG) therapy did not exert the effect on the recurrent fever, whereas fever and skin rash were well controlled by etanercept and a low dose of corticosteroids in our patient. Anti-TNF therapy is effective in suppressing fever, normalizing inflammatory markers, panniculitis, arthritis, decreasing the frequency of blood transfusion, controlling seizures, and improving enteropathy with weaning from parenteral nutrition [5, 8, 9]. However, several patients who were treated with IVIG/SCIG (*n* = 7), anakinra (*n* = 6), corticosteroids (*n* = 3), infliximab (*n* = 1), adalimumab (*n* = 1), and etanercept (*n* = 1), respectively, still exhibited a poor or no response to treatments. Colchicine and thalidomide could control fever in a few cases.

Progressive B-cell deficiency and/or hypogammaglobulinemia resulting from proteotoxic stress and maturation arrest are characteristic features of *TRNT1* mutations. Although long-term use of systemic corticosteroids or disease-modifying anti-rheumatic drugs (DMARDs) rarely induces profound B-cell depletion, these treatments can potentially exacerbate underlying B-cell dysfunction in affected patients and, therefore, require cautious use. In our case with B-cell lymphopenia, high-dose systemic corticosteroids were initiated and subsequently tapered to less than 1 mg/kg/day within one month, followed by complete discontinuation at 15 months of age. Notably, the patient did not develop moderate to severe infections throughout the two-year follow-up period.

Onset age of ≤ 3 months, seizures, and decreased B cell count were significantly associated with a higher mortality rate and poor long-term survival (Supplementary Table S4 and Fig. 5). Specifically, these factors might indicate more severe genetic or functional immunological defects and defects in the mitochondria, as immunometabolism affects T cell, B cell, neutrophil, and macrophage maturation and function [11]. Large-scale prospective cohort studies are needed to find the factors or features associated with prognosis and survival.

Hematopoietic stem cell transplantation (HSCT) was performed in six patients who did not respond to the aforementioned treatment choices. Unfortunately, the role of HSCT remains controversial owing to the high mortality rate of 66.67%. The causes of death in four patients included pulmonary hemorrhage, respiratory failure, sepsis, and multi-organ failure within 3 months post-HSCT. The other two patients who survived post-HSCT still had hearing impairment and retinopathy/pigmentary retinitis, despite remission of systemic inflammation and fever. Several challenges, including avoiding drugs with possible mitochondrial toxicity and patient tolerability to transplant-related complications, are associated with allogeneic HSCT in patients with mitochondrial dysfunction [11, 12].

## Conclusions

The clinical phenotypes associated with *TRNT1* mutations are highly variable. Younger age of onset of ≤ 3 months, seizures, and decreased B-cell count are significant poor prognostic factors for survival. Anti-TNF therapy early in life can stabilize patients with autoinflammatory phenotypes; however, the role of HSCT remains controversial.

## Supplementary Information

Below is the link to the electronic supplementary material.

Supplementary Material 1 (DOCX 65.9 KB)

## Data Availability

The datasets generated and analyzed during the current study are available from the corresponding author on reasonable request.
