# Supplementary material for: Prognostic Factors for Sideroblastic Anemia with B-cell Immunodeficiency, Periodic Fevers, and Developmental Delay Due to TRNT1 Gene Mutations: A Case Report and Systematic Review
Source: J Clin Immunol. 2026 Mar 7;46(1):34. doi: 10.1007/s10875-026-02000-6 (PMC13009118; doi:10.1007/s10875-026-02000-6)
Supplement: Supplementary file 1 — Supplementary Material 1 (DOCX 65.9 KB) [file 10875_2026_2000_MOESM1_ESM.docx]

**Supplementary Table S1.** Laboratory data of the patient in the present study

|  | **Laboratory data** | | **Reference range** |
| --- | --- | --- | --- |
| **Age** | 3 months old | 2 years old |  |
| **Hemogram** |  |  |  |
| White blood cell count, K/μL | 11.47 | 6.75 | 6.0－14.99 (1－6 mo), 4.86－13.51 (6 mo－6 y) |
| Neutrophil, % (count) | 41.9 (4.806) | 34.8 (2.25) | 8.9－76.0 % (1－6 mo), 16.9－74.0 % (6 mo－6 y) |
| Lymphocyte, % (count) | 53.8 (6.171) | 54.2 (3.66) | 30.4－86.7 % (1－6 mo), 18.1－79.9 % (6 mo－6 y) |
| Hemoglobin, g/dL | 11.3 | **9.5** | 8.9－12.7 (1－6 mo), 10.1－12.7 (6 mo－6 y) |
| Mean corpuscular volume, fL | 77.2 | **65.2** | 74.1－96.4 (1－6 mo), 69.5－85.0 (6 mo－6 y) |
| Platelet count, K/μL | **965** | 447 | 229－597 (1－6 mo), 189－459 (6 mo－6 y) |
| **Inflammatory markers** | (highest level) |  |  |
| ESR 1 hr, mm/hr | **58** | 2 | 2－20 |
| C-reactive protein, mg/dL | **>40** | **1.44** | <1 |
| Procalcitonin, ng/mL | **13.21** | **0.769** | <0.5 |
| Interleukin-6, pg/mL | **694.3** | - | <7 |
| TNF-α, pg/mL | **156** | - | <8.1 |
| Ferritin, ng/mL | **3593.44** | **204.59** | 4.63－204.0 |
| **Autoimmune markers** | (highest level) |  |  |
| Anti-nuclear antibodies | **1:80 (+) AC-4** | 1:80 (-) | 1:80 negative |
| C3, mg/dL | 151.38 | 117.1 | 87－200 |
| C4, mg/dL | **59.38** | **86.01** | 19－52 |
| **Serum immunoglobulin levels** |  |  |  |
| IgG, mg/dL | **1443.27**^a^ | 518.3 | 176－581 (2－6 mo), 424－1051 (2 yr) |
| IgA, mg/dL | 22.87 | 15.28 | 4.6－46 (2－6 mo), 14－123 (2 yr) |
| IgM, mg/dL | **20.64** | 54.36 | 24－89 (2－6 mo), 48－168 (2 yr) |
| IgE, IU/mL | 9.09 | - | 0.6－13 (< 1 yr) |
| IgD, IU/mL | <46.7 | - | <100 |
| **Lymphocyte subset** |  |  |  |
| CD3^+^ T cell, % (count, /μL) | 83 (5121) | 82.5 (3018) | 2100－6500 (1－11 mo), 1610－4230 (2－5 yr) |
| B cell, % (count, /μL) | **6 (370)** | **2.4 (88)** | 500－1500 (1－11 mo), 700－1300 (2－5 yr) |
| NK cell, % | 11 | 14 | 6－15 |
| Total CD4^+^ T cell, % (count, /μL) | 51 (3147) | 47 (1720) | 1500－4800 (1－11 mo), 900－2860 (2－5 yr) |
| Naïve CD4^+^ T cell, % | **42.5** | 33 | 21－33 |
| Memory CD4^+^ T cell, % | 8.5 | 14 | 7.8－18 |
| CD3^+^CD8^+^ T cell, % (count, /μL) | 30 (1851) | 33 (1207) | 750－2470 (1－11 mo), 630－1910 (2－5 yr) |
| CD3^+^ TCRγδ^+^ T cell, % | **1.6** | 3.7 | 3.1－12 |
| **Mitogen stimulation lymphocyte proliferation responses** |  |  | (a healthy control) |
| PHA, cpm (% of control) | 118106 (102.11%) |  | 115665 |
| PWM, cpm (% of control) | **24039 (47.91%)** |  | 50171 |
| Anti-CD3/anti-CD28 Ab, cpm (% of control) | 127353 (107.81%) |  | 118126 |

Abbreviations: ESR, erythrocyte sedimentation rate; PHA, phytohemagglutinin; PWM, pokeweed mitogen; *cpm*, counts per minute

^a^IgG level was measured two months after high dose intravenous immunoglobulin therapy. **Abnormal values are shown in bold.**

**Supplementary Table S2. Genetic mutations of *TRNT1* of the patient and the parents**

| **Position** | **Exon**  **(het)** | **Nucleotide change^a^** | **MAF world/TW** | **Patho Score^b^** | **Parent** | **Pathogenicity**  **ClinVar/ACMG** | **Criteria fulfilled in the ACMG guideline for variant interpretation** |
| --- | --- | --- | --- | --- | --- | --- | --- |
| 3:3147471 | 7 (het) | c.824T>A  p.Leu275Ter | NR/NR | NA | Father is het | NA/Pathogenic | PVS1, PM2, PM3, PP4 |
| 3:3148095 | 8 (het) | c.1246A>G  p.Lys416Glu | 0.0006/NR | 6/13 | Mother is het | Pathogenic/Likely Pathogenic | PS1, PM2, PM3, PP4 |

Abbreviations: het, heterozygous; NR, not reported; NA, not available; MAF, minor allele frequency; TW, Taiwan; ACMG, American College of Medical Genetics and Genomics; PVS, pathogenic very strong; PM, pathogenic moderate; PP, pathogenic supporting.

^a^Transcript of *TRNT1* gene: NM_182916.3

^b^Pathogenicity score for missense variants: number of in silico software predicated as deleterious. Software included SIFT, Polyphen2-DVAR, Polyphen2_HDIV, MutationTaster, FATHMM, PROVEAN, MetaSVM, MetaLR, LRT, MutationAssessor, M_CAP, CADD, and fathmmMKL.

**Supplementary Table S3**. Clinical features and genetic mutations in patients with *TRNT1* mutations

| **No** | **Allele 1** |  | **Allele 2** |  | **Type** | **Country/**  **Ethnicity** | **Sex** | **Age^a^ (mo)** | **Decreased B cells** | **Other immune^b^** | **Anemia** | **Fever** | **Dev delay** | **IVIG/**  **SCIG** | **Anti-TNF** | **HSCT** | **Out-come** | **Ref** |
| --- | --- | --- | --- | --- | --- | --- | --- | --- | --- | --- | --- | --- | --- | --- | --- | --- | --- | --- |
| 1 | c.569G>T, p.Arg190Ile | M | c.569G>T, p.Arg190Ile | M | homo (C) | South Asian (Pakistani) | F | 8 | (+) | Low CD4^+^T, CD8^+^T, NK, high inflam | (+) | (+) | (+) | (+) |  |  | D, 14 y | [1, 2, 8] |
| 2 | c.569G>T, p.Arg190Ile | M | c.569G>T, p.Arg190Ile | M | homo (C) | South Asian (Pakistani) | M | 1.5 | (+) | Low T, NK, immature Neu | (+) | (+) | (+) | (+) |  | (+) | A, 8 y | [1, 2, 8] |
| 3 | c.668T>C, p.Ile223Thr | M | c.1057-7C>G | S | comp het | Caucasian | F | 1 | (+) |  | (+) | (+) | (+) | (+) |  |  | D, 5 y | [2, 8] |
| 4 | c.668T>C, p.Ile223Thr | M | c.1057-7C>G | S | comp het | Caucasian | M | 0.75 |  |  | (+) | (+) | (+) |  |  |  | D, 56 mo | [2, 8] |
| 5 | c.668T>C, p.Ile223Thr | M | NA | NA | het | Caucasian | F | 1 | (+) |  | (+) |  | (+) | (+) |  |  | D, 25 mo | [2, 8] |
| 6 | c.218_219ins22 | fs | c.668T>C, p.Ile223Thr | M | comp het | Caucasian | M | 7 | (+) | High inflam | (+) | (+) | (+) | (+) |  |  | D, 16 mo | [2, 8] |
| 7 | c.668T>C, p.Ile223Thr | M | c.668T>C, p.Ile223Thr | M | homo (C) | Caucasian | F | 3 | (+) |  | (+) | (+) | (+) | (+) |  |  | A, 20 y | [2, 8] |
| 8 | c.461C>T, p.Thr154Ile | M | c.497T>C, p.Leu166Ser | M | comp het (C) | Caucasian | F | 2 | (+) | High inflam | (+) |  | (+) |  |  |  | A, 5 y | [2, 8] |
| 9 | c.569G>T, p.Arg190Ile | M | c.569G>T, p.Arg190Ile | M | homo (C) | South Asian (Pakistani) | F | 7 | (+) | Low CD4^+^T, CD8^+^T, Lym | (+) | (+) | (+) |  |  |  | D, 28 mo | [2, 8] |
| 10 | c.668T>C, p.Ile223Thr | M | c.1057-7C>G | S | comp het | Caucasian | M | 1.75 | (+) | High CRP | (+) | (+) | (+) |  |  | (+) | A, 4 y | [2, 8] |
| 11 | c.472A>G, p.Met158Val | M | c.977T>C, p.Ile326Thr | M | comp het | Caucasian | M | 1 | (+) |  | (+) | (+) |  |  |  |  | A, 4 y | [2, 8] |
| 12 | c.461C>T, p.Thr154Ile | M | c.608+1G>T | S | comp het | Caucasian | NA | NA |  |  |  |  |  | (+) |  | (+) | D, 9 mo | [2] |
| 13 | c.del1054_1056+10 | S | c.1246A>G, p.Lys416Glu | M | comp het | Caucasian | NA | NA |  |  |  |  |  | (+) |  |  | A, 15 y | [2] |
| 14 | c.del1054_1056+10 | S | c.1246A>G, p.Lys416Glu | M | comp het | Caucasian | NA | NA |  |  |  |  |  | (+) |  |  | A, 18 y | [2] |
| 15 | c.668T>C, p.Ile223Thr | M | c.1142insATGT, p.Trp381fs | fs | comp het | Afro-Caribbean | NA | NA |  |  |  |  |  | (+) |  |  | A, 12 mo | [2] |
| 16 | c.668T>C, p.Ile223Thr | M | c.1252_1253insA, p.Ser418fs | fs | comp het | Caucasian | NA | NA |  |  |  |  |  | (+) |  |  | A, 6 mo | [2] |
| 17 | c.443C>T, p.Ala148Val | M | c.443C>T, p.Ala148Val | M | homo (C) | Turkish | F | 0.6 |  |  | (+) |  | (+) |  |  |  | D, 21 mo | [4] |
| 18 | c.383A>G, p.Asp128Gly | M | c.518A>T, p.Tyr173Phe | M | comp het | Greek-Cypriot | M | 42 |  |  |  |  | (+) |  |  |  | A, 8 y | [4] |
| 19 | c.126_128delAGA | D | c.1246delA, p.Ser418fs | fs | comp het | Na | M | 12 |  |  | (+/-), micro | (+) |  |  |  |  | A, 19 y | [13] |
| 20 | c.609-26T>C | S | c.1246insA, p.Ser418fs | fs | comp het | Na | M | Child |  |  | (+/-), micro |  |  |  |  |  | A, 21 y | [13] |
| 21 | c.609-26T>C | S | c.1246insA, p.Ser418fs | fs | comp het | Na | M | 156 |  |  | (+/-), micro |  |  |  |  |  | A, 18 y | [13] |
| 22 | c.295C>T, p.Arg99Trp | M | c.295C>T, p.Arg99Trp | M | homo (C) | Indian | F | 9 | (-) |  | (+) | (+) |  | (+) |  |  | A, 15 y | [14] |
| 23 | c.295C>T, p.Arg99Trp | M | c.295C>T, p.Arg99Trp | M | homo (C) | Indian | M | 7 | (-) |  | (+) |  |  | (+) |  |  | A, 10 y | [14] |
| 24 | c.295C>T, p.Arg99Trp | M | c.295C>T, p.Arg99Trp | M | homo (C) | Indian | F | 3 | (-) |  | (+) |  |  | (+) |  |  | A, 9 y | [14] |
| 25 | c.342+5G>T | S | c.668T>C, p.Ile223Thr | M | comp het | Caucasian | F | 0.5 | (+) | Low Neu, PLT, high inflam | (+) | (+) | (+) | (+) |  |  | D, 3 y | [1] |
| 26 | c.342+5G>T | S | c.668T>C, p.Ile223Thr | M | comp het | Caucasian | M | 0.75 | (+) |  | (+) | (+) |  |  |  |  | D, 10 mo | [1] |
| 27 | c.295C>T, p.Arg99Trp | M | c.295C>T, p.Arg99Trp | M | homo (C) | NA | M | 11 | (+) | Low Lym, Neu, NK, High inflam | (+) | (+) | (+) |  |  |  | A, 23 y | [15] |
| 28 | c.1057-7C>G | S | c.1213G>A, p.Gly405Arg | M | comp het | Caucasian | F | 2 | (+) | High inflam | (+) | (+) |  |  |  |  | A, 1 y | [16, 17] |
| 29 | c.608+1G>T | S | c.668T>C, p.Ile223Thr | M | comp het | NA | F | 0 |  |  | (+) |  |  |  |  |  | D, 40 h | [18] |
| 30 | c.608+1G>T | S | c.668T>C, p.Ile223Thr | M | comp het | NA | M | 1 |  |  | (+) |  |  |  |  | (+) | D, 6 mo (day 38 post-HSCT) | [18] |
| 31 | NA | NA | NA | NA | homo | NA | F | NA |  |  | (+) |  |  | (+) |  |  | D, 9 y | [19] |
| 32 | c.495_498del, p.Phe167Thrfs*9 | fs | c.1246A>G, p.Lys416Glu | M | comp het | Latin-America | F | 3 |  | High inflam | (+) | (+) | (+) |  |  |  | A, 6 mo | [20] |
| 33 | c.465T>C, p.Ile155Thr | M | c.608G>A, p.Arg203Lys | M | comp het | Italian | M | 3 | (+) | Low TRECs, KRECs | (+) | (+) | (+) | (+) |  |  | D, 26 mo | [21] |
| 34 | c.1057-7C>G | S | c.1092A>T, p.Glu346Asp | M | comp het | NA | M | 5 |  |  | (+) | (+) | (+) |  |  |  | A, 21 mo | [22] |
| 35 | c.644A>G, p.His215Arg | M | c.644A>G, p.His215Arg | M | homo (C) | Saudi Arabian | F | 1 |  | Low Lym, High inflam | (+) | (+) |  |  |  |  | D, 7 y | [5] |
| 36 | c.644A>G, p.His215Arg | M | c.644A>G, p.His215Arg | M | homo (C) | Saudi Arabian | F | 24 | (-) | High inflam | (+) | (+) | (+) |  | (+) |  | A, 8 y | [5] |
| 37 | c.668T>C, p.Ile223Thr | M | c.488A>T, p.Asp163Val | M | comp het | Mixed European | M | 1 | (+) | Hemophagocytosis, high inflam | (+) | (+) | (+) | (+) |  | (+) | D, 3y (day 92 post-HSCT) | [5] |
| 38 | c.295C>T, p.Arg99Trp | M | c.488A>T, p.Asp163Val | M | comp het | Mixed European | F | 0.75 | (+) | Hemophagocytosis, high inflam | (+) | (+) | (+) | (+) | (+) |  | A, 4 y | [5] |
| 39 | c.295C>T, p.Arg99Trp | M | c.488A>T, p.Asp163Val | M | comp het | Mixed European | F | 1.5 | (+) | Low NK, high inflam | (+) | (+) | (+) | (+) | (+) |  | A, 12 y | [5] |
| 40 | c.329C>T, p.Thr110Ile | M | c.383A>G, p.Asp128Gly | M | comp het | Mixed European | M | 72 | (+) |  | (+) | (+) | (+) | (+) |  |  | A, 26 y | [5] |
| 41 | c.329C>T, p.Thr110Ile | M | c.383A>G, p.Asp128Gly | M | comp het | Mixed European | F | 1 | (+) | Low T | (+) | (+) | (+) | (+) |  |  | D, 9 y | [5] |
| 42 | c.1246A>G, p.Lys416Glu | M | c.1245_1246insA,p.Ser418Lysfs*9 | fs | comp het | Mixed European | M | 2 | (+) | High inflam | (+) | (+) | (+) | (+) |  |  | A, 2 y | [5] |
| 43 | c.668T>C, p.Ile223Thr | M | c.1245_1246insA,p.Ser418Lysfs*9 | fs | comp het | Mixed European | F | 0.75 | (+) | Low T | (+) | (+) | (+) | (+) | (+) |  | A, 39 mo | [5] |
| 44 | NA | NA | NA | NA | NA | NA | M | 0 |  |  | (+) |  |  |  |  |  | A, 10 mo | [23] |
| 45 | NA | NA | NA | NA | NA | NA | M | 0 |  |  | (+) |  |  |  |  |  | A, 22 mo | [23] |
| 46 | c.668T>C, p.Ile223Thr | M | c.668T>C, p.Ile223Thr | M | homo | Portuguese | M | 8 |  | Pancytopenia | (+) | (+) | (+) |  |  |  | A, 18 y | [24] |
| 47 | NA | NA | NA | NA | het | NA | NA | NA |  |  |  | (+) | (+) | (+) |  |  | NA | [10] |
| 48 | c.977T>C,  p.Ile326Thr | M | c.977T>C,  p.Ile326Thr | M | homo (C) | African | F | 0 | (+) | Low CD8^+^T, NK, high inflam | (+), resolved at 4 y | (+) | (+) |  |  |  | A, 4 y | [17] |
| 49 | c.218_219ins22, | fs | c.218_219ins22 | fs | homo | NA | M | 6 |  |  | (+) | (+) | (+) |  |  |  | 2 of the 3 died before 2 y^c^ | [25] ^c^ |
| 50 | c.668T>C, p.Ile223Thr | M | c.829G>T, p.Glu277X | N | comp het | NA | F | 2.4 |  |  | (+) | (+) | (+) |  |  |  |  | [25] ^c^ |
| 51 | c.977T>C, p.Ile326Thr | M | c.977T>C, p.Ile326Thr | M | homo | NA | F | 19 |  |  | (+) |  | (+) |  |  |  |  | [25] ^c^ |
| 52 | c.668T>C, p.Ile223Thr | M | c.1057-7C>G | S | comp het | Caucasian | F | Infancy |  |  | (+) | (+) |  |  |  |  | A,40 y | [26] |
| 53 | c.295C>T, p.Arg99Trp | M | c.1234C>T, p.Arg412X | N | comp het | Japanese | M | 1 | (+) | Low PLT, WBC | (+) | (+) | (+) | (+) |  |  | A, 12 y | [7] |
| 54 | c.448C>T, p.Arg150Cys | M | NA | NA | het | NA | F | 3 |  | High CRP, ESR | (+) | (+) |  |  | (+) |  | A, 5 y | [27] |
| 55 | NA | NA | NA | NA | comp het | China | M | 480 |  |  |  |  |  |  |  |  | A, 40 y | [28] |
| 56 | c.525delT, p.Leu176X | N | c.938T>C, p.Leu313Ser | M | comp het | China | F | 24 | (+) | High inflam | (+) | (+) |  |  |  |  | A, 5 y | [29] |
| 57 | c.498_501delATTT,  p.Phe167fs | fs | c.947C>T, p.Ala316Vall | M | comp het | NA | F | 2 | (+) |  | (-) | (+) | (+) | (+) |  |  | A, 1 y | [30] |
| 58 | c.495_498del, p.Phe167Thrfs*9 | fs | c.1246A>G, p.Lys416Glu | M | comp het | NA | F | 6 | (+) | High inflam | (+) | (+) | (+) | (+) | (+) |  | A, 4y8m | [31] |
| 59 | c.383A>G, p.Asp128Gly | M | c.1168G>A, p.Gly390Ser | M | comp het | Caucasian | M | 4 | (+) | Low NK, high CRP, ferritin | (+) | (+) | (+) | (+) | (+) |  | A, 3 y | [32] |
| 60 | c.361G>A, p.Glu121Lys | M | c.407C>G, p.Ala136Gly | M | comp het | Brazilian | F | 1 | (+) | High DNT, high CRP | (+) | (+) |  | (+) | (+) |  | A, 3 y | [33] |
| 61 | c.608G>A, p.Arg203Lys | M | c.1246A>G, p.Lys416Glu | M | comp het | Italian | F | 0.75 | (+) | High inflam | (+) | (+), | (+) |  | (+) |  | A, 22 y | [9] |
| 62 | c.938delT, p.Leu313fs | fs | c.1246A>G, p.Lys416Glu | M | comp het | Italian | F | 4 |  | High inflam | (+) | (+) | (+) |  | (+) |  | A, 13 y | [9] |
| 63 | c.914A>T, p.Asp305Val | M | c.914A>T, p.Asp305Val | M | homo (C) | Turkish | M | 6 | (-) |  | (+) |  |  | (+) |  |  | A, 16 y | [34] |
| 64 | c.88A>G, p.Met30Val | M | c.363G>T, p.Glu121Asp | M | comp het | China | M | 6 |  | High CRP | (+) | (+) |  |  |  |  | A, 3.5 y | [35] |
| 65 | c.302T>C, p.Ile101Thr | M | c.1234C>T, p.Arg412X | N | comp het | China | M | 12 |  | High CRP | (+) |  | (+) |  |  |  | A, 12 y | [35] |
| 66 | c.295C>T, p.Arg99Trp | M | c.295C>T, p.Arg99Trp | M | homo | Spanish | M | 14 |  |  | (+/-), micro | (+) |  | (+) |  |  | A, 49 y | [36] |
| 67 | c.1057-7C>G | S | c.1092A>T, p.Glu346Asp | M | comp het | Asian/European | F | 8 | (-) |  | (-) | (+) | (+) |  |  |  | A, 8 y | [37] |
| 68 | c.1057-7C>G | S | c.1092A>T, p.Glu346Asp | M | comp het | Asian/European | M | 12 | (-) |  | (+) | (+) | (+) |  |  |  | A, 5 y | [37] |
| 69 | c.1056+1G>A | S | c.1246A>G, p.Lys416Glu | M | comp het | China | F | 8 | (-) | High CRP | (+) | (+) | (+) |  |  |  | A, 16 y | [38] |
| 70 | c.1056+1G>A | S | c.1246A>G, p.Lys416Glu | M | comp het | China | M | 4 | (-) | High CRP | (+) | (+) | (+) | (+) |  |  | A, 8 y | [38] |
| 71 | c.574C>T, p.Gln192X | N | c.464T>C, p.Ile155Thr | M | comp het | China | F | 3 | (+) | High inflam | (+) | (+) |  | (+) | (+) | (+) | D, 17 mo, (day 17 post-HSCT) | [38] |
| 72 | c.706G>A, p.Glu236Lys | M | c.706G>A, p.Glu236Lys | M | homo | China | M | 1.5 | (+) | High NK, High inflam | (+) | (+) | (+) | (+) |  |  | D, 1 y | [39] |
| 73 | c.88A>G, p.Met30Val | M | c.907>G, p.Gln303Glu | M | comp het | China | F | 0 | (+) | low PLT, High inflam | (+) | (+) |  | (+) | (+) |  | A, 5 y | [39] |
| 74 | c.443C>T, p.Ala148Val | M | c.692C>G, p.Ala231Gly | M | comp het | China | M | 6 | (-) |  | (+) |  | (+) |  |  |  | A, 3 y | [40] |
| 75 | c.824T>A, p.Leu275X | N | c.1246A>6, p.Lys416Glu | M | comp het | Taiwan | F | 0.25 | (+) | High inflam | (+/-), micro | (+) | (+) | (+) | (+) |  | A, 21 mo | Current case |

Abbreviations: *M*, missense; *S*, splicing; *fs*, frameshift; *N*, nonsense; *D*, deletion; *dev*, developmental; *NA*, not available; *homo*, homozygous; *het*, heterozygous; *comp het*, compound heterozygous; *C*, consanguinity; *T*, T cells; *NK*, Natural killer cells; *DNT*, double-negative T cells; *PLT*, platelet; *Lym*, lymphocytes; *Neu*, neutrophils; *micro*, microcytosis; *TRECs*, T-cell receptor excision circles; *KRECs*, kappa-deleting recombination excision circles; *inflam*, inflammatory markers; *CRP*, C-reactive protein; *ESR*, erythrocyte sedimentation rate; *D*, died; *A*, alive; *IVIG*, intravenous immunoglobulins; *SCIG*, subcutaneous immunoglobulins; *HSCT*, hematopoietic stem cell transplantation; *TNF*, tumor necrosis factor

^a^Age of onset (month old)

^b^Other immunological or hematological abnormalities

^c^This study reported 2 of the 3 patients died before 2 years of age, but which patient died was not documented

Supplementary Table S4. The correlation between mortality and B-cell deficiency according to mutation types

| **Mutation types** | **Death^a^** | ***P* value^b^** | **B cell deficiency** | ***P* value^b^** |
| --- | --- | --- | --- | --- |
| Missense (N=100) | 25/96 (26.04%) | 0.241 | 54/70 (77.14%) | 0.557 |
| Nonsense (N=4) | 2/4 (50.00%) |  | 0/3 (00.00%) |  |
| Frameshift (N=13) | 1/12 (8.33%) |  | 0/5 (00.00%) |  |
| Splicing (N=19) | 7/19 (36.84%) |  | 5/9 (55.56%) |  |

^a^Two patients with mortality were not included in the analysis due to unclear clinical characteristics.

^b^Analyzed by chi-square test.

N=allele number

Supplementary Table S5. Clinical features in patients with and without mortality

|  | **Alive**  **(N=52)** | **Died^a^**  **(N=19)** | ***P* value** |
| --- | --- | --- | --- |
| Demographic features |  |  |  |
| Female | 23/48 (47.92%) | 11/18 (61.11%) | 0.340 |
| Onset age ≤3 months | 22/47 (46.81%) | 14/17 (82.35%) | 0.021 |
| Clinical features |  |  |  |
| Recurrent fever | 36/46 (78.26%) | 14/18 (77.78%) | 1.000 |
| Hearing loss | 20/46 (43.48%) | 4/18 (22.22%) | 0.114 |
| Seizures | 3/46 (6.52%) | 7/18 (38.89%) | 0.004 |
| Laboratory data |  |  |  |
| Anemia | 39/46 (84.78%) | 18/18 (100%) | 0.177 |
| Decreased B cell number | 22/32 (68.75%) | 12/12 (100%) | 0.041 |
| Hypogammaglobulinemia | 32/35 (91.43%) | 14/14 (100%) | 0.548 |
| Treatment |  |  |  |
| HSCT | 2/52 (3.85%) | 4/19 (21.05%) | 0.040 |

Abbreviations: HSCT, hematopoietic stem cell transplantation

^a^Two patients with mortality were not included in the analysis since their clinical characteristics were unclear.
